# Supplementary material for: Biological iron-sulfur storage in a thioferrate-protein nanoparticle
Source: Nat Commun. 2017 Jul 20;8:16110. doi: 10.1038/ncomms16110 (PMC5524996; doi:10.1038/ncomms16110)
Supplement: Supplementary Information [file ncomms16110-s1.pdf]

SI GUIDE

File Name: Supplementary Information
File Description: Supplementary Figures, Supplementary Methods and Supplementary References.

File Name: Peer Review File
File Description:

Supplementary Methods

XAS data acquisition and analysis. Iron K-edge XAS data were collected on solutions of IssA in 50 mM Tris buffer in the presence of 30% glycerol, frozen in 1 mm × 3 mm × 23 mm acrylic sample cuvettes closed with metal free kapton adhesive tape. Data were collected using the Stanford Synchrotron Radiation Lightsource (SSRL) structural molecular biology XAS beamline 7-3, employing a Si(220) double-crystal monochromator with harmonic rejection by setting the collimating mirror cut-off to 9 keV. Incident and transmitted X-ray intensities were monitored using N_2-filled gas ionization chambers with a sweeping voltage of 1.8 kV, and X-ray absorption was measured as the iron $K_{\alpha 12}$ fluorescence excitation spectrum using an array of 30 germanium detectors equipped with a manganese filter and a Soller slit assembly. During data collection, samples were maintained at a temperature of approximately 10 K using an Oxford instruments liquid helium flow cryostat. For each iron K-edge data set, eight scans each of 40 min. duration were accumulated, and the energy was calibrated by reference to the absorption of a metallic iron foil measured simultaneously with each scan, assuming a lowest energy K- edge inflection point of 7,111.3 eV. The energy threshold of the extended X-ray absorption fine structure (EXAFS) oscillations (k = 0 Å^{-1}) was assumed to be 7130.0 eV. Data were collected over the extended *k*-range to a maximum *k* of 18.2 Å^{-1}, in order to obtain the best resolution.

Sulfur K-edge XAS data were collected on frozen solutions using an Oxford Instruments helium cryostream apparatus at 20 K, yielding an estimated sample temperature of 20-50 K. The presence of the chlorine K-edge at 2,833 eV meant that the sample needed to be prepared in the absence of added chloride and sulfonate based buffers, and samples were contained in acrylic cuvettes closed with 6 µm thick polypropylene windows to minimize attenuation of the X-ray beam. Sulfur K-edge XAS data were collected on SSRL beamline 4-3, using a Si(111)

monochromator and rejection of Si(333) and higher harmonics was achieved by setting the angle of the upstream vertically collimating mirror to give a high energy cutoff of ~5 keV. The incident X-ray intensity was measured with a helium filled gas ionization chamber with a sweeping voltage of 1 kV, and total fluorescence was measured by using a nitrogen-filled Stern-Heald-Lytle fluorescence ion chamber detector (The EXAFS Co., Pioche NV, USA). The incident X-ray energy was calibrated by reference to the lowest K-edge energy peak of a sodium thiosulfate ($Na_2S_2O_3$) standard, assuming a peak energy of 2,469.2 eV as previously described[1]. The energy threshold of the extended X-ray absorption fine structure (EXAFS) oscillations ($k = 0$ $Å^{-1}$) was assumed to be 2,475.0 eV. The high X-ray cross sections at the low X-ray energies of the sulfur K-edge mean that samples in such experiments are particularly prone to radiation damage[2]. The near-edge portion of the spectrum was monitored for changes indicative of radiation damage and only small changes were observed (Supplementary Figure 8). Sixteen scans each of 30 minutes duration from three different samples were averaged to obtain a final data set, with careful screening of data to remove scans with irreproducible features with a maximum number of 8 scans per sample. Sulfur K-edge EXAFS data were collected to a maximum k of 13.5 $Å^{-1}$ because of truncation due to the presence of residual atmospheric argon which has a K-absorption edge at 3,205.9 eV. For both iron and sulfur K-edge data the program XAS collect was used to collect data[3].

Expression and purification of the apo-IssA construct using *E. coli*. The gene encoding IssA (PF2025) was amplified by PCR using *Pyrococcus furiosus* DSM 3638 genomic DNA isolated by ZymoBead Genomic DNA Kit (Zymo Research) and a set of primers, sense (5'-GGGCATATGAAGATAGCGATCCCAACTAATGGAGGAGG-3') and anti-sense (5-

GGGCTCGAGAGTTGCTACTTTAATTGCCTCTTCAACTGGAG-3'). Nde I and Xho I restriction enzyme sites were designed on the sense and anti-sense primers, respectively. The N-terminal 107 residues of PF2025 were amplified in order to remove the IssA tail in the recombinant form. The amplified DNA fragment and pET-24a(+) vector (Novagen) were digested by Nde I and Xho I and ligated by T4 DNA ligase (New England Biolabs). The assembled plasmid was transformed into XL1-blue E. coli competent cells for sequence confirmation. The plasmid was then transformed into the BL21-CodonPlus (DE3)-RIPL strain (Agilent Technologies) for protein expression. The *E. coli* transformant was grown at 37°C in 2xYT medium with 20 µg/ml chloramphenicol and 50 µg/ml kanamycin to an OD_{600} of 0.6-0.8. The protein expression was induced with 300 µM IPTG and switched to 25°C for 16 to 18 hours. Cells were harvested by centrifugation at 10,000 x *g* for 10 minutes and resuspended in 50 mM Tris, 300 mM NaCl, 2 mM DTT, pH 8.0 (buffer A) and complete protease inhibitor (Roche) at a ratio of 1 g wet cell wt. to 3 ml buffer. All the purification steps were carried out under anaerobic condition. Cells were lysed by 0.5 mg/ml lysozyme with stirring for 1 hour at room temperature. The mixture was frozen at -20°C overnight and thawed at room temperature for 2 hours. DNase I (0.002%, Sigma) was then added and stirred for 1 hour. After centrifugation at 7,500 rpm for 10 minutes to remove cell debris and insoluble material, the supernatant was collected and applied to a column containing Ni Sepharose 6 Fast Flow (GE Healthcare Life Sciences), which was equilibrated with buffer A. The column was washed with buffer A and the adsorbed protein was eluted with 500 mM imidazole in buffer A. Protein purity was examined by SDS-PAGE.

Phylogenetic analysis and structural model. Metagenomic sequences and identical (duplicate) sequences were removed from the 5041 sequences containing the IPR003731 domain, and the

resulting 4630 sequences were aligned using Clustal Omega, version 1.2.1, with the default parameters[4]. TrimAl[5] was used to remove multiple alignment positions with greater than 99.78% gaps (i.e. 10 or fewer sequences had sequence content at that position), and the resulting alignment was used to construct a maximum likelihood phylogenetic tree using IQ-Tree[6] (version 1.5.3). The IQ-Tree standard model selection test was used to automatically determine the best-fit model (WAG+G4: general amino acid matrix with a discrete Gamma model). This model tree was refined using ultrafast bootstrap approximation (UFBoot) and Shimodaira–Hasegawa-like approximate likelihood ratio tests with 1000 bootstrap replicates for each. iTol[7] was used for analysis and display of the phylogenetic tree. For visualization and clade selection, branch lengths were not used (e.g. cladogram) and branches with bootstrap confidence values less than 70% were removed. The COPid[8,9] server was used for amino acid analysis and pI was calculated with a custom program based on the ExPASy Compute pI/Mw algorithm[8,9]. The Phyre 2.0[10] server was used to thread model the IssA sequence on the homologous *M. thermautotrophicus* structure (PDB ID 1EO1).

All peptides queried (19)

PF number	ORF number	InterPro name	MW (Da)	Expect value	Peptide matches	Percent coverage
**PF2025**	18978397	**IssA**	18990	**0.0047**	5	27.9
PF1027	18977399	putative RNA methylase	43494	3.9	6	16.1
PF0938	18977310	aconitase/3-isopropylmalate dehydratase large subunit	46181	4.6	4	15.3
PF0193	18976565	ABC transporter-like	35846	5.4	3	21.6
PF0738	18977110	methyltransferase type 11	21023	7.7	4	18.2
PF1604	18977976	cystathionine beta-synthase, core	17979	7.9	3	25.2
PF0920	18977292	metal-dependent phosphohydrolase, HD region	25773	9.7	3	15.7
PF1095	18977467	NULL	11453	12	2	37.9
PF1612	18977984	NULL	9806	12	2	39.5
PF1459	18977831	L-fucose isomerase-like	54272	14	4	8.5
PF1335	18977707	hydroxyethylthiazole kinase	28309	15	3	15.1
PF1379	18977751	probable translation factor pelota	40482	15	4	16.6
PF0209	18976581	lysine biosynthesis enzyme LysX	30991	15	3	12.1
PF0236	18976608	phosphoribosyl pyrophosphokinase	30928	16	3	14.7
PF1807	18978179	ribosomal protein L32e	15525	19	2	28.5
PF1128	18977500	CRISPR-associated protein, TM1793	36293	22	3	18
PF2015	18978387	DNA/RNA helicase, C-terminal	86885	24	6	11
PF0475	18976847	initiation factor 2B related	31078	31	3	13
PF1896	18978268	NULL	28483	31	3	9.5
PF1345	18977717	ribonuclease Z	35105	35	3	16

Supplementary Table 1. Analysis of IssA immunoprecipitate for additional binding proteins. The top twenty protein hits are shown from the resulting peptide masses after analysis using MASCOT software, searching a *Pyrococcus furiosus*-specific database, and allowing for 2 missed cleavages and a mass difference of ± 0.4 Da. An expectation value of 0.05 or less is significant; significant expectation values are highlighted in bold.

Element	Atoms per IssA monomer
Fe	38.73 ± 3.60
Zn	1.33 ± 0.13
Cu	0.68 ± 0.06
Mg	0.24 ± 0.08
Co	0.14 ± 0.01

Supplementary Table 2. Elemental composition of IssA purified from *Pyrococcus furiosus*. Values are from ICP-MS data and are expressed as mole atom/mole IssA monomer, assuming pure IssA. Shown are the metals out of fifty-five measured by ICP-MS that gave values ≥ 0.1. Error bars indicate standard deviation of two independent ICP-MS runs.

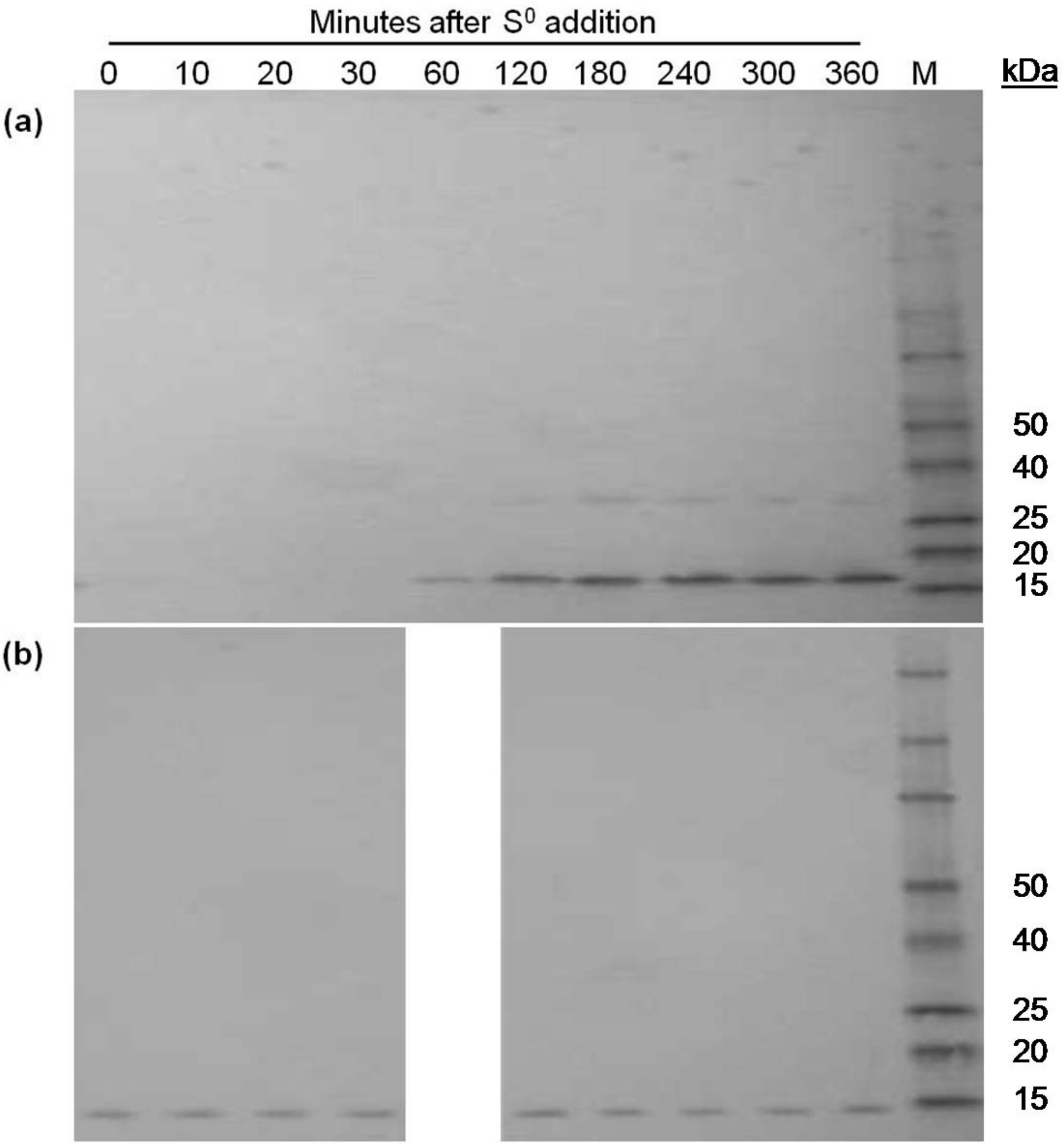

Supplementary Figure 1. Western blot analysis of whole cell extract from *P. furiosus* grown on maltose medium after S^0 addition at mid-log phase. Lanes are 0, 10, 20, 30, 60, 120, 180, 240, 300, and 360 minutes after S^0 addition. (a) IssA antibody, (b) SOR antibody as a loading control. M: protein standard marker.

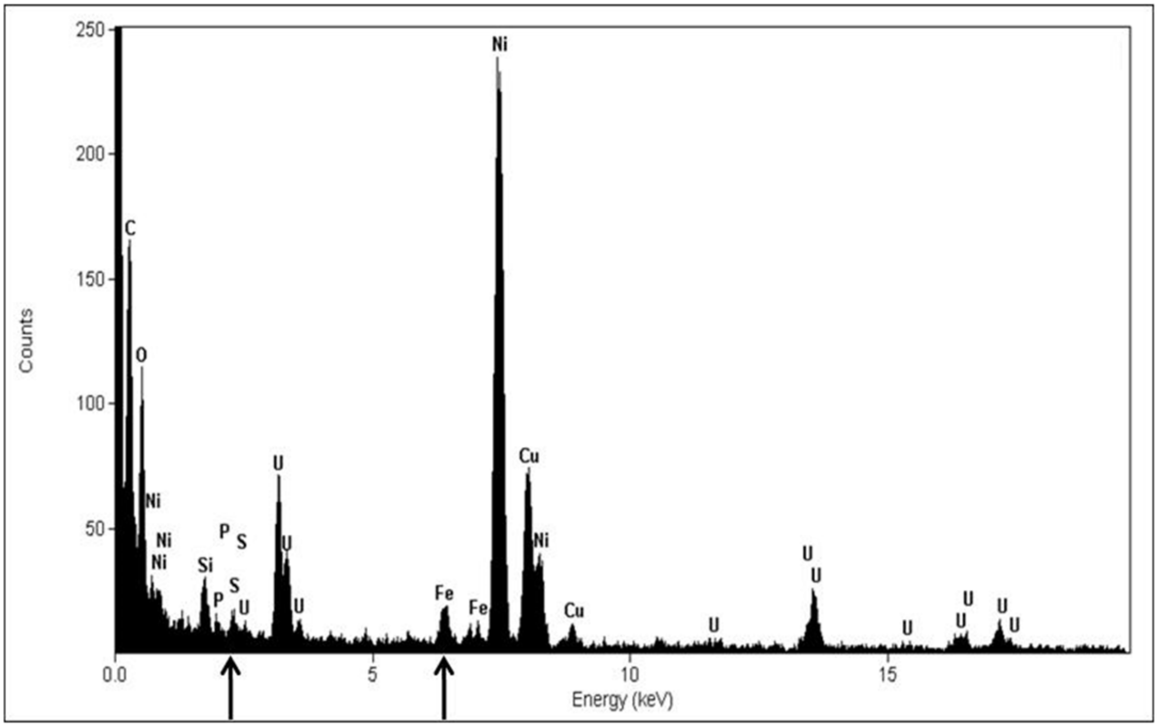

Supplementary Figure 2. Energy dispersive X-ray analysis of an electron dense particle associated with IssA. Element names are noted; nickel and copper are from the support grid and uranium was used to stain the cells. Arrows indicate the Kα energies of S at 2.47 keV and Fe at 7.11 keV.

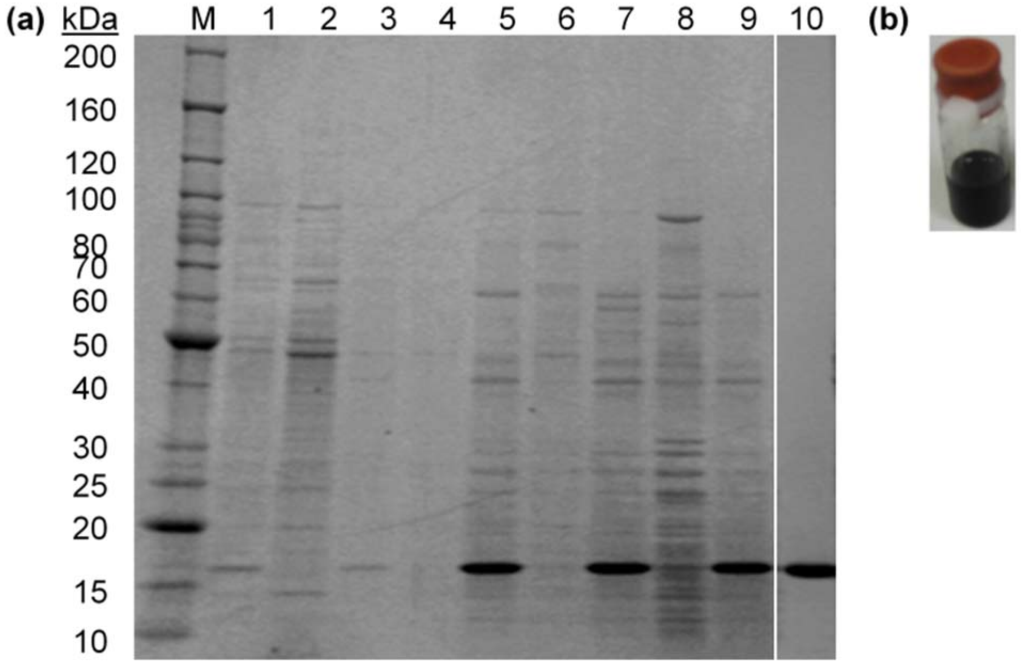

Supplementary Figure 3. IssA purification from *Pyrococcus furiosus*. (a) SDS-PAGE gel showing IssA purification from cells grown on maltose with 2 g/L S^0; IssA runs at approximately 17 kDa and was confirmed by MALDI-TOF mass spectrometry. M: protein size markers (Invitrogen); lane 1: whole cell extract; lane 2-3: 100,000x*g* supernatant, pellet; lane 4-5: 1% SDS-treated supernatant, pellet; lane 6-7: first wash supernatant, pellet; lane 8-9: second wash supernatant, pellet; lane 10: final IssA sample after CsCl gradient and concentration. (b) Purified IssA is black in color.

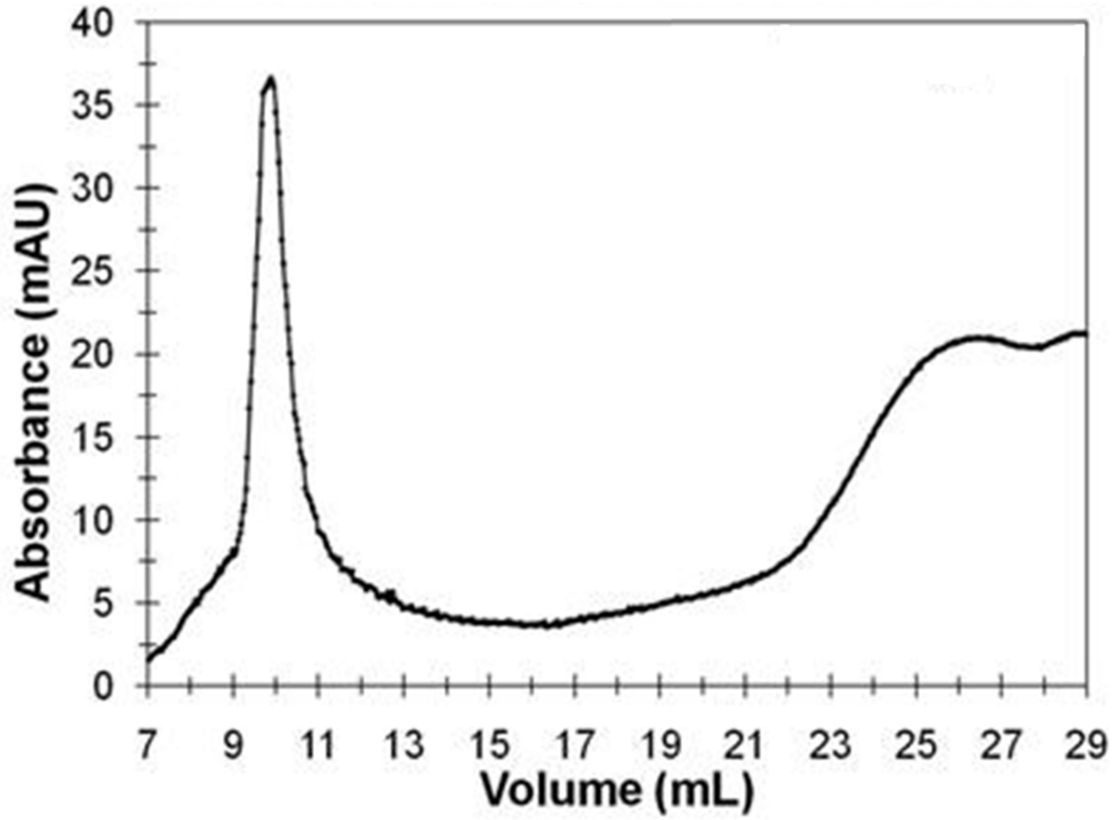

Supplementary Figure 4. IssA molecular weight determination by size exclusion chromatography. The molecular weight of IssA was estimated using analytical column chromatography (Sephacryl S-1000 SF) pre-equilibrated with 50 mM TrisHCl (pH 8.0), 300 mM NaCl, 1 mM DTT. Native IssA from *P. furiosus* eluted near the exclusion limit (100 MDa dextran).

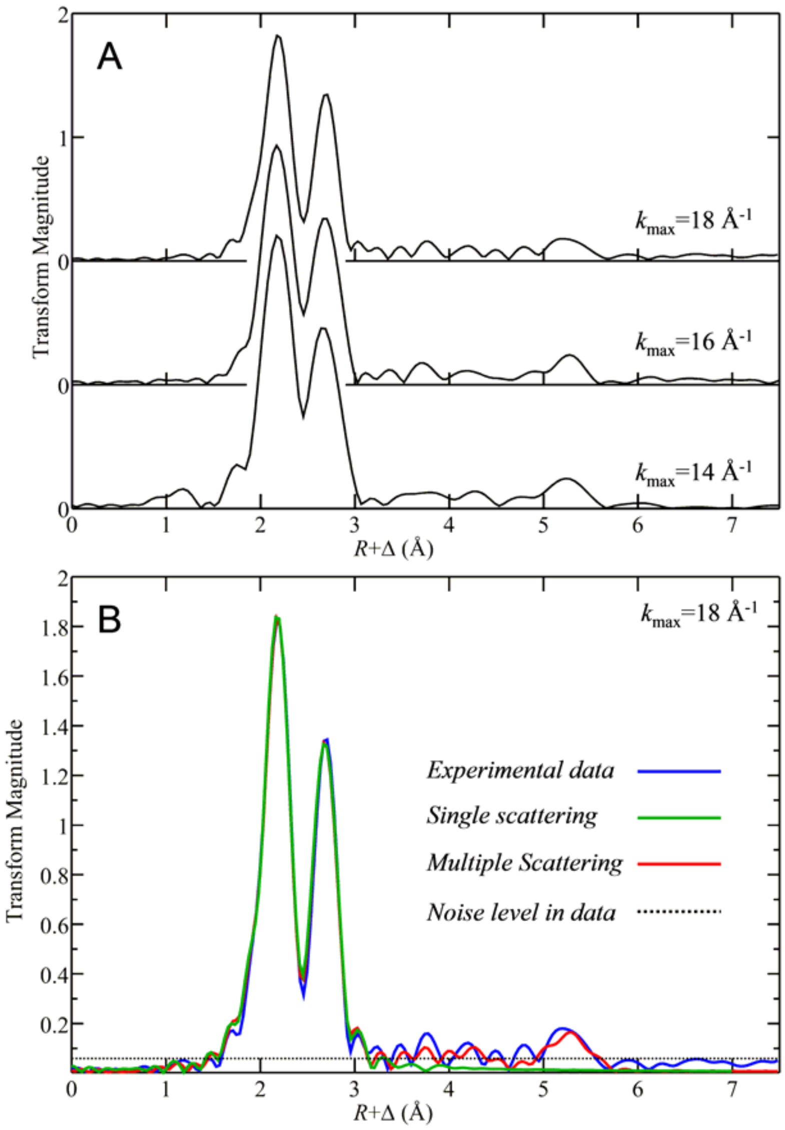

Supplementary Figure 5. Detailed analysis of 5.4 Å iron EXAFS peak. A) Shows EXAFS Fourier transforms (Fe—S phase-corrected) for different *k*-ranges showing persistence of the 5.4 Å transform peak. **B)** Shows the EXAFS Fourier transform of the high-*k* data (18 Å^{-1}) (blue line) modeled using multiple scattering (red line) or single scattering (green line) plus the estimated noise level in the data at this *k*-range (broken line). We note that the EXAFS noise level is expected to be "white" with equal amplitudes at all frequencies but that the noise at very low frequency will be effectively removed by the EXAFS spline function, which is why the apparent Fourier transform noise level falls below the broken line at low *R* values.

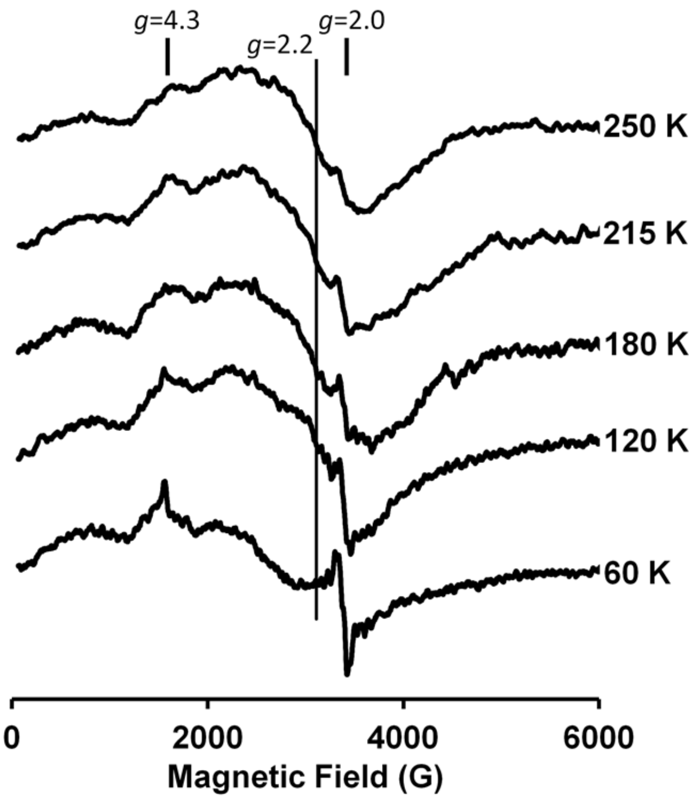

Supplementary Figure 6. Temperature-dependence of the EPR spectra of IssA. The spectra were recorded under non-power-saturating conditions at a microwave frequency of 9.60 GHz, with a modulation amplitude of 6.4 G and a microwave power of 20 mW. Broad scans at various temperatures show a very broad signal centered around g = 2.2 (marked by the long vertical line) that increases in intensity with increasing temperature due to antiferromagnetic coupling in the thioferrate chains. The positions at g = 4.3 and g = 2.0 are marked. The former is indicative of trace adventitiously-bound high-spin (S = 5/2) Fe^{3+} or magnetically isolated linear $[3Fe\text{-}4S]^{+}$ clusters (S = 5/2). The origin of the weak signal in the g = 2 region is unknown.

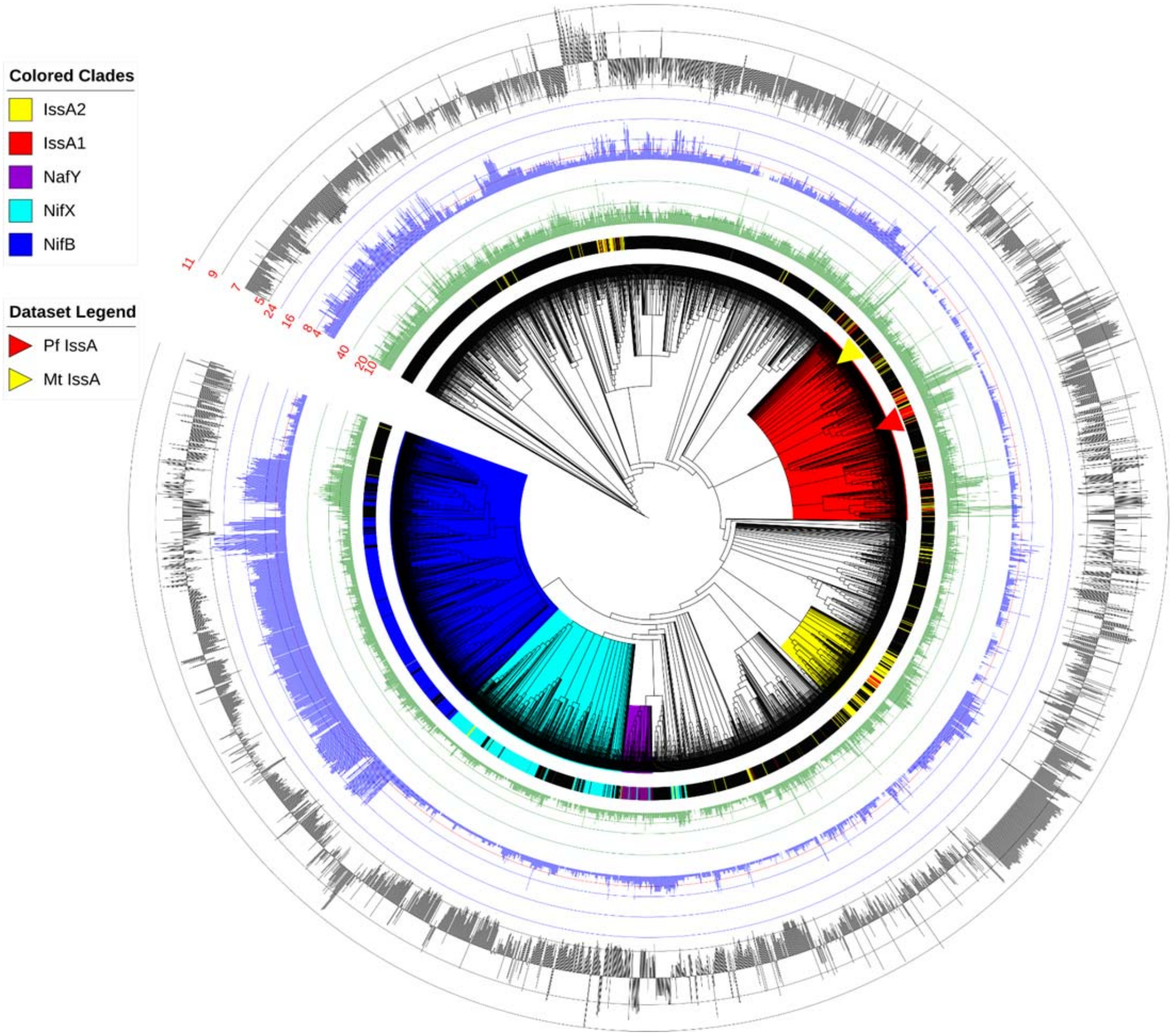

Supplementary Figure 7. Cladogram of proteins containing the IPR003731 domain. Colored clades contain members of the NifB (IPR005980, blue), NifX (IPR013480, turquoise), and NafY (IPR031763, yellow) InterPro protein families. The proposed IssA clade based on *P. furiosus* IssA (red) contains member proteins (red lines on innermost ring) with pI ≥ 9, fewer than 5 cysteines and at least 20% glycine in the C-terminal 40 residues, similar to IssA including the C-terminal tail. The IssX clade (78% confidence; yellow) shares some of these features, but is more distantly related to *P. furiosus* IssA and contains member proteins (yellow lines on innermost ring) with pI ≥ 8, fewer than 10 cysteines and at least 10% glycine residues in the last 40 amino acids. Many members of this clade appear to contain a shortened version of the *P. furiosus* IssA C-terminal tail. Blue, green and purple lines on the innermost ring mark individual members of IPR005980, IPR013480 and IPR031763, respectively. The second ring (green) plots the percentage of glycine in the last 40 residues. The third ring (blue) plots the number of cysteines in the protein where the red scale line shows 4 cysteines. The outer ring (black) plots predicted protein pI centered at pH 7.0. *P. furiosus* IssA and the *Methanothermobacter thermautotrophicus* homolog (MTH1175) used to predict the structure of the *P. furiosus* N-terminal domain (Fig. 6a), are shown by red and yellow arrowheads, respectively.

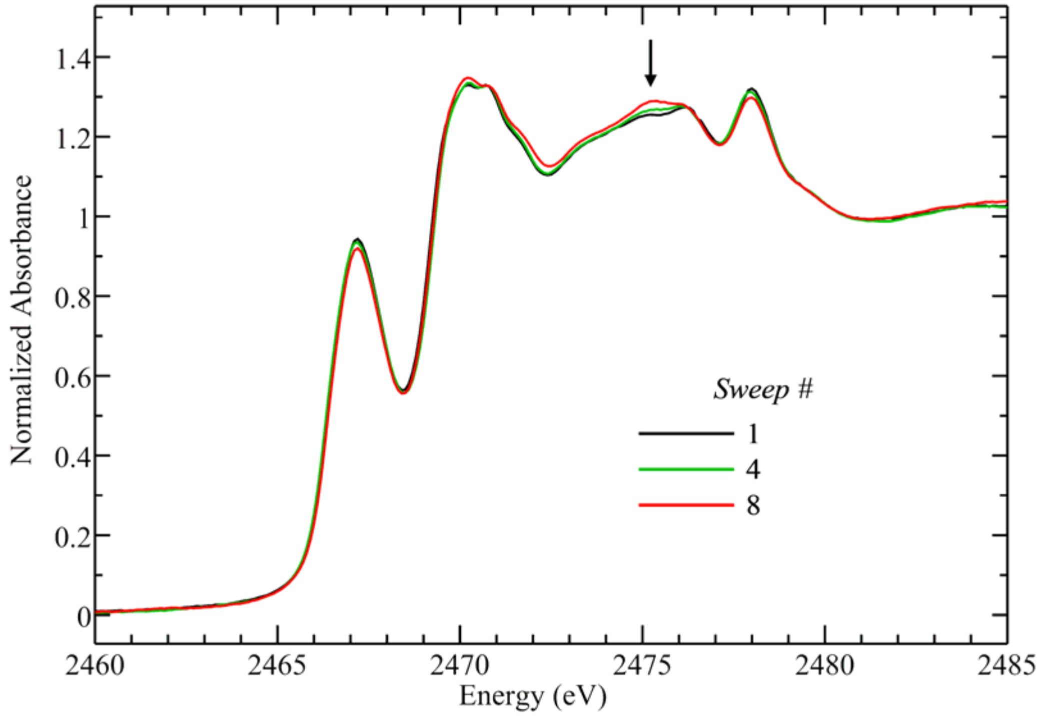

Supplementary Figure 8. Effects of extended X-ray beam exposure on the sulfur K-edge XAS spectrum of a single IssA sample. Spectra are shown for the first scan, the fourth scan and the eighth scan with each scan taking approximately 30 min. Only subtle changes in the near-edge spectrum are observed in this sample and these are most pronounced at around 2475 eV (indicated by the arrow), suggesting that photo-oxidation of a small fraction of the sample has occurred.

Supplementary References

1. Pickering, I. J., Prince, R. C., Divers, T. & George, G. N. Sulfur K-edge X-ray absorption spectroscopy for determining the chemical speciation of sulfur in biological systems. *FEBS Lett.* **441**, 11-14 (1998).
2. George, G. N. *et al.* X-ray-induced photo-chemistry and X-ray absorption spectroscopy of biological samples. *J. Synchrotron Radiat.* **19**, 875-886 (2012).
3. George, M. XAS-Collect: a computer program for X-ray absorption spectroscopic data acquisition. *J. Synchrotron Radiat.* **7**, 283-286 (2000).
4. Sievers, F. *et al.* Fast, scalable generation of high-quality protein multiple sequence alignments using Clustal Omega. *Mol. Syst. Biol.* **7** (2011).
5. Capella-Gutiérrez, S., Silla-Martínez, J. M. & Gabaldón, T. trimAl: A tool for automated alignment trimming in large-scale phylogenetic analyses. *Bioinformatics* **25**, 1972-1973 (2009).
6. Minh, B. Q., Nguyen, M. A. T. & von Haeseler, A. Ultrafast Approximation for Phylogenetic Bootstrap. *Mol. Biol. Evol.* **30**, 1188-1195 (2013).
7. Letunic, I. & Bork, P. Interactive Tree Of Life v2: Online annotation and display of phylogenetic trees made easy. *Nucleic Acids Res.* **39**, W475-478 (2011).
8. Gasteiger, E. *et al.* in *The Proteomics Protocols Handbook* (ed John M. Walker) 571-607 (Humana Press, 2005).
9. Bjellqvist, B. *et al.* The focusing positions of polypeptides in immobilized pH gradients can be predicted from their amino acid sequences. *Electrophoresis* **14**, 1023-1031 (1993).

10. Kelley, L. A., Mezulis, S., Yates, C. M., Wass, M. N. & Sternberg, M. J. E. The Phyre2 web portal for protein modeling, prediction and analysis. *Nat. Protocols* **10**, 845-858 (2015).
